# Effective CRISPR/Cas9-mediated correction of a Fanconi anemia defect by error-prone end joining or templated repair

**DOI:** 10.1038/s41598-018-36506-w

**Published:** 2019-01-25

**Authors:** Henri J. van de Vrugt, Tim Harmsen, Joey Riepsaame, Georgina Alexantya, Saskia E. van Mil, Yne de Vries, Rahmen Bin Ali, Ivo J. Huijbers, Josephine C. Dorsman, Rob M. F. Wolthuis, Hein te Riele

**Affiliations:** 1 Division of Tumor Biology and Immunology, The Netherlands Cancer Institute, Plesmanlaan 121, 1066 CX Amsterdam, The Netherlands; 2 Section of Oncogenetics, Department of Clinical Genetics, Cancer Center Amsterdam, Amsterdam University Medical Centers, De Boelelaan 1118, 1081 HV Amsterdam, The Netherlands; 3 Mouse Clinic for Cancer and Aging research (MCCA) Transgenic Facility, The Netherlands Cancer Institute, Plesmanlaan 121, 1066 CX Amsterdam, The Netherlands; 4 Genome Engineering Oxford, Sir William Dunn School of Pathology, University of Oxford South Parks Road, OX1 3RE, Oxford, UK

## Abstract

Fanconi anemia (FA) is a cancer predisposition syndrome characterized by congenital abnormalities, bone marrow failure, and hypersensitivity to aldehydes and crosslinking agents. For FA patients, gene editing holds promise for therapeutic applications aimed at functionally restoring mutated genes in hematopoietic stem cells. However, intrinsic FA DNA repair defects may obstruct gene editing feasibility. Here, we report on the CRISPR/Cas9-mediated correction of a disruptive mutation in *Fancf*. Our experiments revealed that gene editing could effectively restore *Fancf* function via error-prone end joining resulting in a 27% increased survival in the presence of mitomycin C. In addition, templated gene correction could be achieved after double strand or single strand break formation. Although templated gene editing efficiencies were low (≤6%), FA corrected embryonic stem cells acquired a strong proliferative advantage over non-corrected cells, even without imposing genotoxic stress. Notably, Cas9 nickase activity resulted in mono-allelic gene editing and avoidance of undesired mutagenesis. In conclusion: DNA repair defects associated with FANCF deficiency do not prohibit CRISPR/Cas9 gene correction. Our data provide a solid basis for the application of pre-clinical models to further explore the potential of gene editing against FA, with the eventual aim to obtain therapeutic strategies against bone marrow failure.

## Introduction

Fanconi anemia is a rare genetic disease characterized by developmental defects, growth retardation, bone marrow failure, and cancer predisposition. To date, 22 distinct FA genes have been identified. The gene products operate in a genomic maintenance pathway that promotes replication fork stability and homology-directed repair (HDR), conferring resistance to endogenous aldehydes and interstrand crosslinking (ICL) agents like mitomycin C (MMC)^1–4^. On a molecular level, FANCM and associated sensor proteins detect DNA replication stress, resulting in the recruitment of the FA core complex (FANCA, -B, -C, -E, -F, -G -L and FA-associated proteins). The core complex acts as an E3 ligase that activates FANCD2 and FANCI by placing a single ubiquitin residue on both of these proteins resulting in replication fork progression towards the DNA lesion and coordination of DNA repair involving the SLX4 (FANCP) scaffold protein, ERCC4 (FANCQ) nuclease, translesion synthesis protein MAD2L2 (FANCV), and the homology-directed repair machinery (BRCA2 (FANCD1), PALB2 (-N), RAD51C (-O), RAD51 (-R), BRCA1 (-S), and XRCC2 (-U))^1^.

A bone marrow transplantation (BMT) is currently the only effective treatment for bone marrow failure in FA patients. Although recent advances have significantly enhanced success rates, BMTs correlate with an increased risk of developing squamous cell carcinoma (SCC) in young FA adults due to the toxic effects of the conditioning and immunosuppression regimens^5–7^.

Recently, gene therapy trials have started to enroll FA-A patients to correct hematopoietic stem cell (HSC) defects by inserting a functional *FANCA* gene using lentiviruses^8,9^. Nevertheless, viral integrations carry oncogenic risks and the effects of long-term ectopic expression of *FANCA* is unknown. In contrast to insertional gene therapy, gene editing offers the opportunity to correct FA-causing mutations in patient-derived cells^10–13^. Using *ex-vivo* gene editing and autologous transplantation, FA corrected HSCs could potentially rescue the bone marrow phenotype of patients without harsh conditioning regiments. Importantly, case reports suggest that naturally occurring FA gene correction, observed in mosaic patients, results in improved hematopoiesis and that correction of a single HSC may suffice to restore normal hematopoiesis^14–18^.

The discovery and implementation of the clustered-regularly-interspaced-short-palindromic-repeats (CRISPR) and CRISPR-associated protein 9 (Cas9) gene editing technology potentially provides the tools to target and correct the majority of mutations found in FA patients as long as protospacer adjacent motif (PAM)-defining sequences are present nearby the target sites^10–12,19^. Opportunities to optimize gene editing strategies are offered by selection of the single guide RNA (sgRNA) to target either the transcribed or non-transcribed DNA strand, Cas9 variants to induce DSBs or single-strand breaks (SSBs), and a variety of single or double-stranded donor templates for homology-directed repair (HDR)^20–25^. The cell type in which the gene editing is performed may also influence success rates and DNA repair outcomes, likely due to differences in cell cycle progression, active DNA repair pathways, target site chromatin conformation, and clonal expansion capabilities^26,27^. After a targeted nuclease creates a DNA break, the prevailing cellular DNA repair pathway determines the outcome of the gene editing process. DSB lesions are predominantly repaired by error-prone end joining in which the loose DNA ends are processed and ligated together, resulting in the formation of typical insertions and deletions (indels) at Cas9 target sites^10–12^. Error-free repair of DSBs requires a homologous donor template and is, in principle, ideally suited to correct mutations associated with genetic diseases. Within the canonical homology-directed repair (HDR) pathway, there are distinct mechanisms such as DSB repair via Holliday junction resolution or dissolution, annealing driven strand synthesis, and single-strand DNA incorporation^21–23,25,28^. After DSB formation, single-stranded oligodeoxyribonucleotide (ssODN)-templated repair is suggested to predominantly occur by annealing driven strand synthesis^23,25^. It was also noted that Cas9D10A induced SSBs are a substrate for annealing driven strand synthesis when the ssODN is complementary to the nicked strand, while single-strand DNA incorporation is suggested to be the main repair mechanism when the ssODN has the same polarity as the nicked strand^22,23^. Although gene editing with a nickase may result in lower template-mediated gene editing frequencies than with a double-strand nuclease, DNA nicks are associated with lower levels of erroneous repair^10,21,23^.

Since FA genes are critical to maintain genomic stability and are involved in canonical homologous recombination (*BRCA1/2*, *PALB2*, *RAD51C*, *RAD51*, and *XRCC2*), the question has been raised whether template-based gene editing can be successfully performed in FA-deficient cells^12^. Moreover, Richardson *et al*. recently stated that several FA and FA-associated genes (*FANCA*, -*D2*, -*E*, -*F*, -*L*, -*T*, and *USP1*) are required for efficient templated repair of Cas9-induced DSBs using a ssODN^29^.

Initial gene editing efforts involving FA have relied upon a Zinc Finger Nuclease (ZFN) to insert a *FANCA* expression cassette flanked by homology arms into the *AAVS1* safe-harbor locus of patient-derived primary fibroblasts and CD34^+^ cells. While this HDR approach successfully complemented cellular FA defects and avoided gene therapy risks associated with random viral integrations, it still relied on ectopic FA gene expression with possible deleterious effects^30,31^.

Osborn *et al*. used CRISPR/Cas9 to correct a c.456 +4 A > T mutation in the FA core complex gene *FANCC*, but were successful only after simultaneously complementing the FA defect and including a puromycin cassette to select for templated HDR events^32^. Osborne *et al*. also reported the functional correction of a c.1461 A > T *FANCI* mutation in human induced pluripotent stem cells (iPSCs) using Cas9D10A nickase and a DNA plasmid as donor template, achieving HDR frequencies of 66% after three rounds of MMC selection^27^. Furthermore, Kramarzorva *et al*. corrected a c.886DelGT mutation in *FANCD1*/*BRCA2* patient-derived primary fibroblasts using CRISPR/Cas9 double-strand break formation and ssODN repair templates with a frequency of ~23% after selection by PARP-inhibitors^33^. The requirement for selection regimens and success in only certain cell types in these reports illustrate that inherent FA DNA repair defects may impair gene editing. Nonetheless, FA cells with defects in *FANCA*, *FANCD1* and *FANCI* seem permissive to templated gene editing using DSBs or single-strand nicks as repair substrates and integrase-defective lentiviral vectors, ssODN, or plasmid donor templates.

Here, we set out to analyze gene editing efficiencies after CRISPR/Cas9-induced DSB or SSB formation in FA-deficient cells. Considering that many FA patients carry alleles with small nucleotide alterations, we opted for a ssODN as the preferred donor for templated repair.

Previously, we generated mouse embryonic stem cells (mESCs) with a four-nucleotide insertion in the *Fancf* gene on one allele of chromosome 7^34^. This c.828insTAAA mutation is predicted to disrupt the open reading frame (ORF) and result in a premature truncation of the FANCF protein, removing a functionally important carboxy terminal interaction domain^35^. FANCF has been shown to function as a central adapter protein in FA core complex formation by interacting with FANCM and the FANCA/FANCG and FANCC/FANCE subcomplexes^35–37^. Therefore, cellular DNA repair defects following *FANCF* inactivation are likely representative for other FA core complex deficiencies, which are the most prevalent in FA patients^38^.

Our data reveal that the pathogenic c.828insTAAA mutation in *Fancf* could be corrected efficiently by CRISPR/Cas9 gene editing following error-prone end joining as well as HDR with a ssODN template. While templated gene editing after nickase activity was less efficient than templated DSB repair, nick repair was rarely associated with errors and predominantly affected only one allele. Although templated gene editing frequencies were low, corrected mESCs showed a dramatic growth advantage with respect to FANCF-defective cells, even without imposing the selective pressure of crosslinking agents, facilitating the recovery of successful gene editing events.

## Results

### A *Fancf* mouse model to assess CRISPR/Cas9 gene editing activity

**Figure 1:**
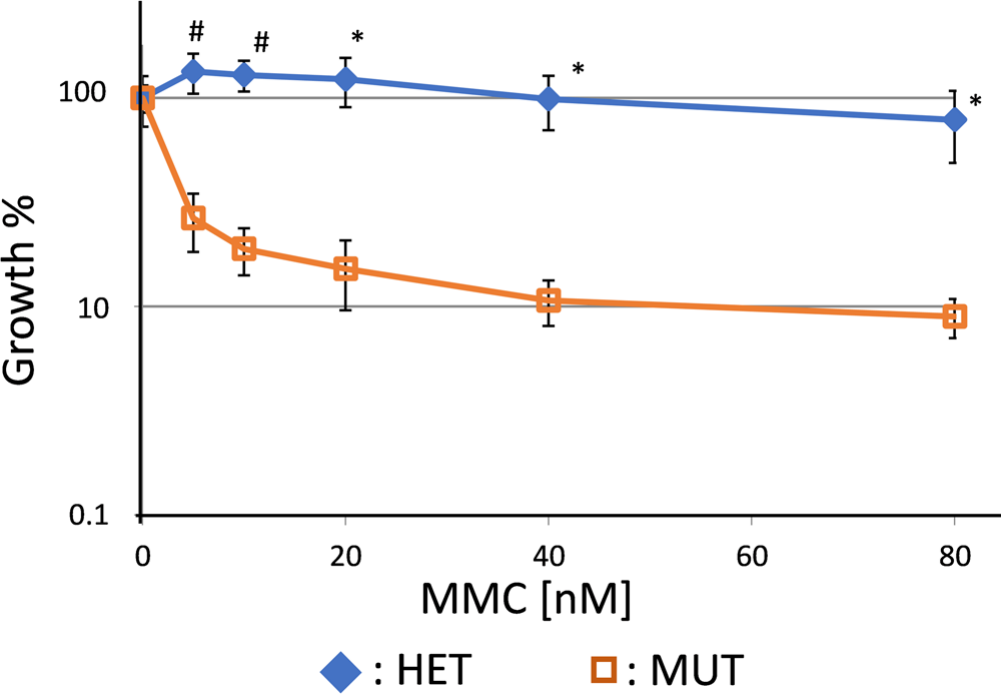
MMC sensitivity in *Fancf* c.828InsTAAA mutant fibroblast. Fibroblasts from *Fancf* c.828insTAAA mutant mice display the FA-characteristic hypersensitivity to MMC, HET = heterozygote, MUT = mutant (n = 2, one sided Student’s T-test, ^#^p ≤ 0.01, *p < 0.05).

Based on embryonic stem cells carrying one *Fancf* allele with an insertion of four nucleotides (TAAA) at position 828 of the coding sequence, a novel transgenic FA mouse model was established and spontaneously immortalized mouse ear fibroblasts were derived. To confirm loss of *Fancf* gene function, homozygous mutant and heterozygous c.828insTAAA fibroblasts were exposed to the crosslinking agent MMC. The mutant fibroblasts displayed the FA-characteristic hypersensitivity to MMC in the growth-inhibition assay, confirming loss of *Fancf* function (Fig. 1).

Mouse embryonic stem cells (ESCs) with bi-allelic c.828insTAAA mutations were derived from blastocysts obtained by crossing heterozygous *Fancf* mice. Of the initial 68 ESC lines that were established, PCR genotyping revealed only 4 (6%) as homozygous mutant, while 36 (53%) were heterozygous, and 28 (41%) wildtype. Since homozygous mutant c.828insTAAA mice were born following Mendelian frequencies (Suppl. Table 1), these numbers suggest a strong selection bias against *in vitro* cultured *Fancf* mutant ESCs (p = 0.0092 Fisher exact test (FET)). Two independent homozygous mutant ESC lines were confirmed by Sanger sequencing and selected for further experiments. The advantage of documenting CRISPR/Cas9 gene editing in cells carrying bi-allelic c.828InsTAAA mutations is that gene corrections by both mutagenic end joining and template-mediated repair can be determined simultaneously.

### CRISPR/Cas9 gene editing activity in *Fancf* mutant mouse ear fibroblasts

**Figure 2:**
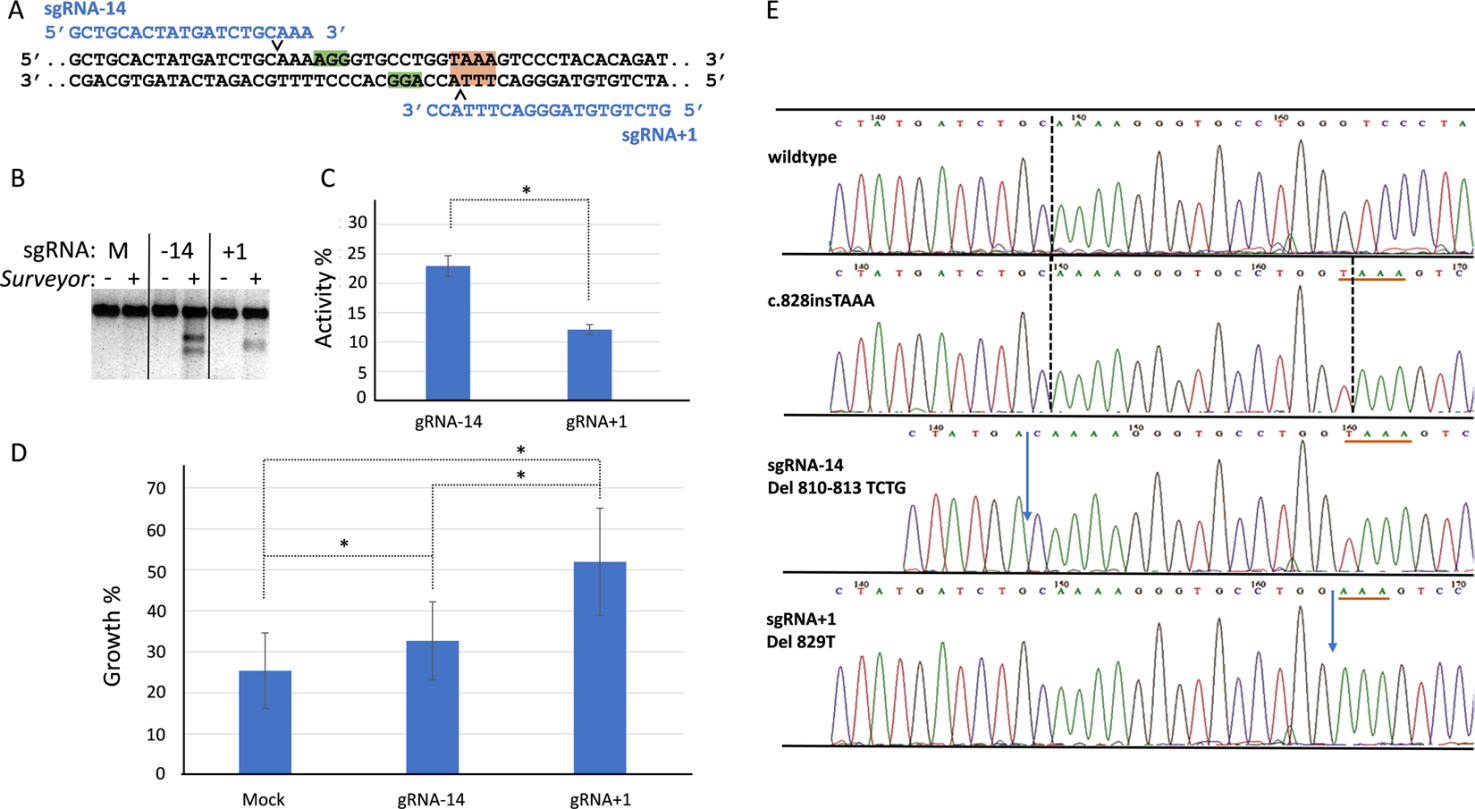
CRISPR/Cas9 and error-prone repair activities restore *Fancf* function. (**A**) Design of sgRNAs to target *Fancf* c.828InsTAAA allele. Analysis of the *Fancf* c.828insTAAA DNA sequence revealed two opposing protospacer sequences (blue) with adjoining PAM motifs (green boxes) with predicted Cas9 nuclease activity sites at positions −14 (^∨^) and + 1(^∧^) of the c.828insTAAA mutation (Red box). (**B**) Surveyor assay indicates error-prone repair activity at targeted positions only when sgRNAs are present as measured by cleavage of heteroduplex DNA by the Surveyor nuclease. (**C**) Quantification of DNA cleavage by Surveyor (n = 3, one sided Student’s T-test, *p = 0.04). (**D**) Error-prone DNA end joining repair correlates with enhanced clonal survival in the presence of 15 nM MMC after double-strand break formation by Cas9 and sgRNA-14 or sgRNA + 1. (n = 3, one sided Student’s T-test, *p ≤ 0.04). (**E**) *Fancf* sequence analysis of single alleles from MMC-selected clones revealed rescued in-frame ORFs following DSB repair by mutagenic end joining. Del810–813 TCTG neutralizes the TAA stop codon at position 829, but also results in amino acid substitutions 271–276 LQKGAW > KRVPGK (see Suppl. Table 2A). While del829T restores the ORF, the FANCF peptide sequence contains an additional Lysine at position 279 (see Suppl. Table 2B). The location of the *Fancf* c.828insTAAA mutation is underlined in red. Vertical dotted lines indicate predicted Cas9 DSB sites. Blue arrows indicate the position of the deletions. Error bars represent standard error.

To establish whether gene editing could be accomplished in the vicinity of the gene-disabling 4 nucleotide insertion in the *Fancf* open reading frame, two distinct sgRNAs were designed (Fig. 2A). sgRNA-14 was predicted to target Cas9 nuclease activity 14 nucleotides (nt) upstream of the pathogenic TAAA insertion, and sgRNA + 1 was designed to bring Cas9 activity directly to the c.828insTAAA insertion. FANCF-deficient fibroblasts were transiently transfected with a modified pX330 expression vector encoding the sgRNA, wildtype (wt)Cas9, and a puromycin resistance marker. After puromycin selection and recovery, robust Surveyor activity was observed in cell pools with an average Surveyor DNA cleavage of 23% for sgRNA-14, and 12% for sgRNA + 1, indicative of random changes induced by error-prone DSB repair (Fig. 2B,C).

Next, cells from the pools were subjected to a colony survival assay in the presence of the crosslinking agent mitomycin C (MMC). Even though MMC was toxic to the majority of mock-transfected cells, 25% of mock gene edited fibroblasts still formed colonies. However, 33% of cells transfected with sgRNA-14, and 52% of cells transfected with sgRNA-1 formed colonies. These data suggest that Cas9 DSB induction and subsequent mutagenic end joining restored *Fancf* gene function and enhanced clonal growth rates in 8% (sgRNA-14, p = 0.04) and 27% (sgRNA + 1, p = 0.02) of the cells (Fig. 2D).

To analyze sequence alterations in the *Fancf* locus after gene editing, single MMC-resistant colonies were picked from the colony survival assay and on average 6 single allele sequence reads were obtained per cell clone (Fig. 2E/Suppl. Table 2). In 56% of the cell clones, three or more distinct *Fancf* alleles were identified, indicating polyploidy, a known consequence of mouse fibroblast immortalization^39^. Remarkably, all analyzed cell clones displayed CRISPR/Cas9-modified alleles, with only 6 out of 39 clones retaining a copy of the parental allele. The low frequency of parental alleles (8.8% for sgRNA-14, 4.2% for sgRNA + 1) underscores the high efficiency of the Cas9 nuclease and error-prone repair in our assay (Suppl. Table 2A,B).

From the 12 analyzed cell clones edited with sgRNA-14, six (50%) contained a *Fancf* allele with a restored ORF. For sgRNA-14, ORF restoration resulted from a short stretch of 6 to 8 amino acid deletions and substitutions not matching the original FANCF polypeptide. Given the low frequency of MMC survival (8%), such ORF-restoring alterations did not necessarily restore FANCF activity (Suppl. Table 2A). 21 (78%) of the 27 analyzed clones that arose after gene editing with sgRNA + 1 showed at least one allele with a restored *Fancf* open reading frame. These ORF-restoring alterations mostly resulted in one or two amino acid substitutions and/or insertions, however, in two clones wildtype *Fancf* alleles were created by gene editing and error-prone repair representing 2.8% of observed alleles (Suppl. Table 2B). Given the higher frequency of MMC survival (27%), subtle ORF-restoring alterations apparently had a higher chance of restoring FANCF activity. Notably, among the 39 fibroblast clones analyzed, we identified four clones (7.8% of encountered alleles) with unrelated sequence insertions at the gene editing locus, involving pX330-Puro plasmid sequences and the *Akt2* locus, 24 Mb away from *Fancf*. This highlights the gene editing risk of inducing partial plasmid integrations or translocations with a gene without a predicted off target site.

### Template-based gene editing in *Fancf* mutant fibroblasts

**Figure 3:**
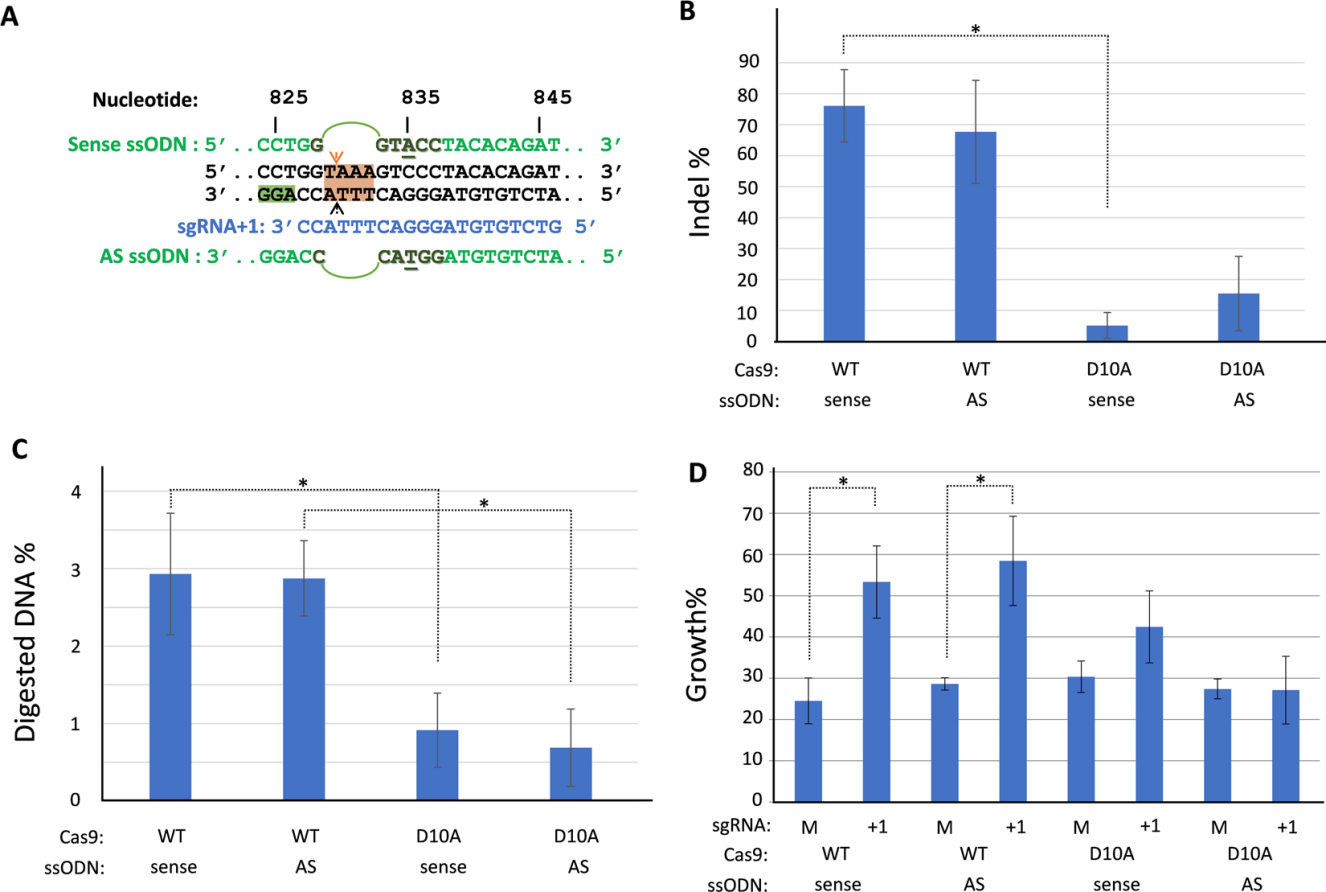
CRISPR/Cas9 templated gene editing analysis in mouse fibroblasts. (**A**) Overview of the sgRNA + 1 target site with sections of the sense and antisense (AS) ssODN that remove the c.828TAAA insertion and introduce a *Kpn*I recognition site by a silent C > A substitution. Predicted cut site for Cas9 are indicated by “^˄^” and “”, with “” indicating the strand that is nicked by Cas9D10A. Nucleotide numbers refer to the mutant c.828InsTAAA *Fancf* gene. (**B**) Overall gene editing efficiencies obtained in mouse fibroblasts depicted as general indel frequencies determined by TIDER (n = 3, one sided Student’s T-test, *p = 0.02). (**C**) Restriction fragment length polymorphism (RFLP) analysis by *Kpn*I digest of the *Fancf* locus provides evidence for template-based repair after Cas9, sgRNA + 1 and ssODN transfection (n = 3, one sided Student’s T-test, *p < 0.03). (**D**) Mouse fibroblasts show enhanced clonal survival in the presence of 12.5 nM MMC after double-strand break formation by Cas9 and sgRNA + 1 independent of the orientation of the HDR template. No significant differences in relative survival were observed after Cas9 nickase activity (n = 3, one sided Student’s T-test, *p < 0.03). Error bars represent standard error, “M” = mock sgRNA.

*Fancf* mutant fibroblasts were transfected with the sgRNA + 1 gene editing plasmid as described above. A 120 nucleotide ssODN in either sense or anti-sense (AS) orientation was co-transfected with the plasmid to serve as donor template to allow precision gene editing by HDR. In addition to correcting the c.828InsTAAA mutation in *Fancf*, the ssODN was designed to introduce a *Kpn*I restriction site by a silent C > A substitution at position 835 of the c.828insTAAA allele (Fig. 3A). To determine whether templated gene editing could also be supported by a targeted single-strand break, parallel experiments were performed with the Cas9D10A nickase, which should only cut the coding (upper) strand (Fig. 3A). Gene editing events in the cell pools were identified by tracking of insertion and deletions (indels) composition (TIDER), while applying a reference wildtype *Fancf* sequence chromatogram to identify del829–832 TAAA gene correction events^40^. According to TIDER, expression of wildtype Cas9 and co-transfection of sense or antisense ssODN templates gave rise to gene editing in respectively 76.1% and 67.7% of total cell pool DNA (Fig. 3B). Indel formation was also detected after gene editing with Cas9D10A, albeit at much lower levels than observed after DSB formation (p ≤ 0.07, sense ssODN). Although the orientation of the ssODN appeared to influence indel formation following Cas9D10A activity (AS > sense), the observed difference was not significant (p = 0.159). TIDER was not able to significantly detect wildtype *Fancf* sequences or minus 4 deletion events in the sequence chromatograms obtained from cell pools, suggesting that c.del829–832TAAA gene correction events were below the detection limit of the assay.

Next, frequencies of templated gene editing were determined in the transfected fibroblast pools by restriction fragment length polymorphism (RFLP) combined with DNA concentration measurements of digestion products using a Tapestation 2200. Positive *Kpn*I digests were not observed in the negative control samples transfected with a ssODN and a non-targeting sgRNA. In contrast, the application of wildtype Cas9 with sgRNA + 1 and ssODN repair template resulted in almost 3% of the cell pool DNA being positive for *Kpn*I digestion, with either orientation of the transfected ssODNs (p ≤ 0.027). After the application of Cas9D10A nickase, 0.9% (sense ssODN) or 0.7% (AS ssODN) of the total cellular DNA showed evidence of a *Kpn*I site within the *Fancf* locus, although due to experimental variation these low RFLP levels did not significantly differ from mock controls. These data also indicate that templated repair after DSB formation was more than 3-fold higher than after single-strand break (SSB) induction (p ≤ 0.029) (Fig. 3C).

To determine whether the observed gene editing events actually resulted in functional rescue of the FA genome maintenance pathway, transfected fibroblasts were exposed to 12.5 nM MMC in clonal survival assays. Fig. 3D indicates that approximately 30% of the cells had gained significant MMC resistance following gene editing with wildtype Cas9, irrespective of the orientation of the ssODN (p ≤ 0.026). The Cas9 D10A nickase plus sense ssODN application, associated with templated repair via single-strand DNA incorporation^23^, showed a non-significant 12% increase in MMC surviving colonies (p = 0.136), while the AS ssODN, a substrate for annealing-dependent strand synthesis^23^, did not show a differential growth effect at all. Overall, these data suggest that *Fancf* correction in gene edited fibroblasts was mostly a consequence of error-prone DNA repair, although HDR with ssODNs was detectable in the cell pools by *Kpn*I RFLP, albeit at low frequencies.

### *Fancf* gene editing efficiencies and off-target analysis in mouse embryonic stem cells

**Figure 4:**
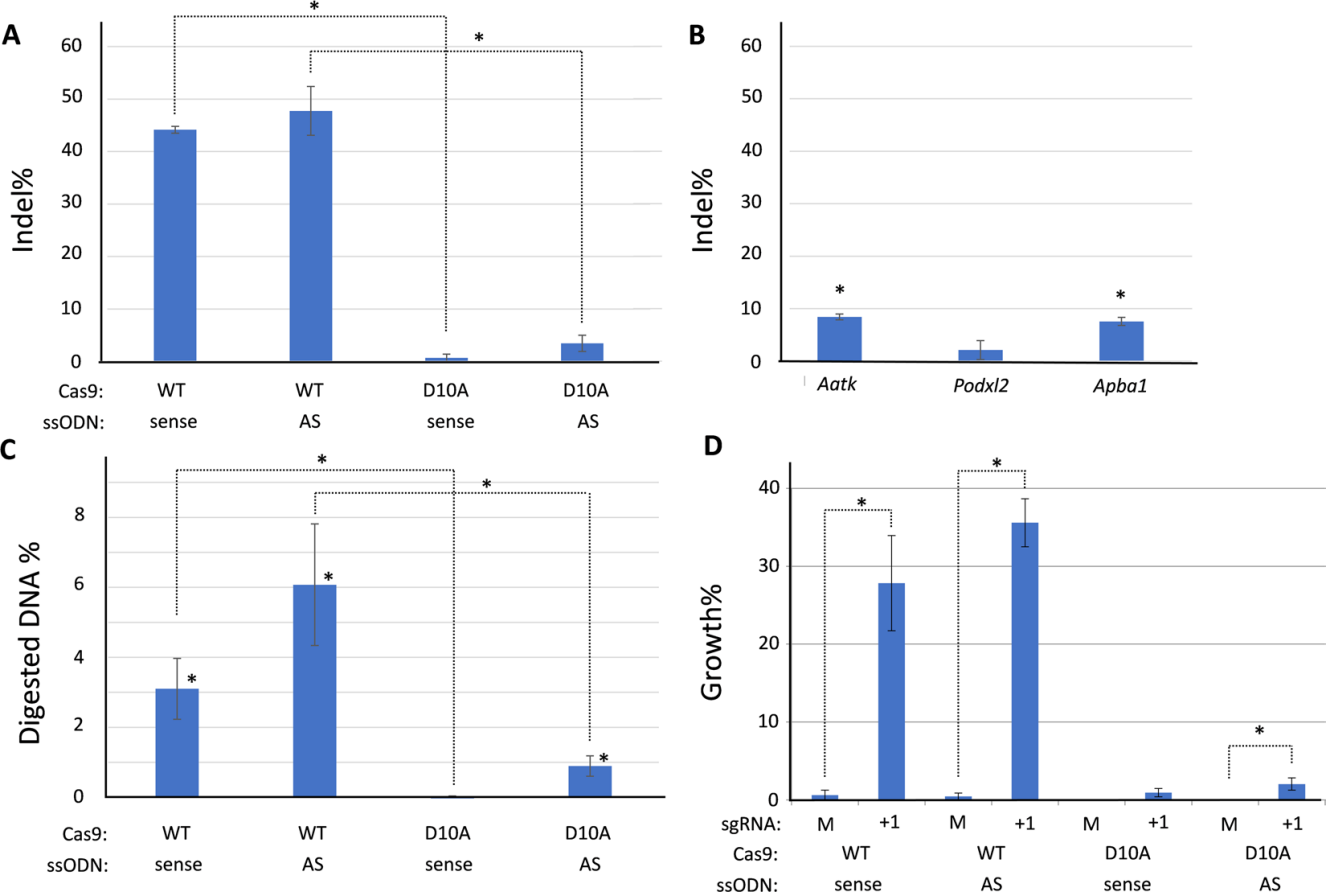
CRISPR/Cas9 templated gene editing analysis in mouse ES cells. (**A**) Overall gene editing efficiencies obtained in mouse embryonic stem cells depicted as general indel frequencies determined by TIDER (n = 3, one sided Student’s T-test, *p ≤ 0.02). (**B**) TIDE analysis of indel formation at predicted off-target loci after wtCas9 and sgRNA + 1 exposure irrespective of the ssODN orientation (n = 3, one sided Student’s T-test, *p ≤ 0.009). (**C**) Restriction fragment length polymorphism (RFLP) analysis by *Kpn*I digest of the *Fancf* locus provides evidence for template-based repair after Cas9 transfection (n = 6, one sided Student’s T-test, *p ≤ 0.02). (**D**) Mouse ES cells show enhanced clonal survival in the presence of 12.5 nM MMC after double-strand break formation by Cas9 and sgRNA + 1 independent of the orientation of the HDR template. A significant difference in relative mESC clone survival was also observed with the antisense (AS) ssODN following Cas9 nickase activity (n = 6, one sided Student’s T-test, *p ≤ 0.025), “M” = mock sgRNA.

To determine templated gene editing frequencies in a cell model considered to be highly proficient in homologous recombination^26,41^, we used mouse embryonic stem cells (ESCs) with bi-allelic c.828insTAAA mutations. ESCs were transiently transfected with pX330-Puro sgRNA + 1 or a mock control plasmid in addition to sense or AS ssODNs for templated repair. Parallel experiments substituting wildtype Cas9 for Cas9D10A nickase were also performed. Cell pools were harvested and gene editing frequencies were determined by TIDER (Fig. 4A). After DSB formation, the observed indel frequencies were 44.1% (sense ssODN) and 47.8% (AS ssODN) (R^2^ ≥ 0.90). In contrast, indel frequencies were much lower when Cas9D10A nickase was used: 0.7% and 3.5% in the presence of sense or antisense ssODNs, respectively (R2 ≥ 0.99).

Considering the observed total on-target indel frequencies mediated by wtCas9 and sgRNA + 1, we investigated whether DNA alterations also occurred at predicted off-targets. Three predicted intragenic off-target sites were selected for analysis displaying 1 or 3 mismatches with sgRNA + 1 (Table 1). DNA from mouse ESC pools treated with wildtype Cas9 and sgRNA + 1 with high frequencies of on-target indel formation were subjected to TIDE analysis at potential off-target sites in *Aatk*, *Podxl2*, and *Apba1*. A representative overview with Sanger sequence chromatograms of on-target and off-targets sites from the same experiment used in the TIDE analysis is depicted in Suppl. Fig. 1. TIDE analysis indicated indel formation at predicted off-targets in *Aatk* and *Apba1* amounting to 8.4% (p = 0.003) and 7.6% (p = 0.009), respectively, while analysis of the *Podxl2* locus did not reveal apparent off-target activity above TIDE background levels (2.2%, p = 0.48) (Fig. 4B). Indel formation at predicted off-target sites with 1 or 3 mismatches in the 5′ area of sgRNA + 1 target sequence was at least 5-fold lower compared to the intended target (p < 0.007).

**Table 1:** Predicted off-target sites for sgRNA + 1.

Gene	Chr	Target sequence	PAM
sgRNA + 1			GTCTGTGTAGGGACTTTACC
On-target:	Fanconi anemia group F *(Fancf)*	7	A------------------- AGG
Off-target	Apoptosis-associated tyrosine kinase (*Aatk*)	11	G---C--------------- CA-
Off-target	Podocalyxin-like 2 (*Podxl2*)	6	-AG-------------G--- T--
Off-target	Amyloid β precursor protein binding (*Apba1*)	19	GC----CA------------ -A-

The similarity between the sequences of the sgRNA + 1 coding sequence and on and off target sites is depicted. Additional information on putative off target genes is supplied in the supplemental data. Dashes indicate identical nucleotides between the sgRNA + 1 coding sequence and genomic DNA.

### Template-based gene editing in *Fancf* mutant mouse embryonic stem cells

Next, *Kpn*I RFLP analysis was performed on DNA from the treated cell pools to determine frequencies of template-based *Fancf* gene correction. With wildtype Cas9, *Kpn*I digestion signatures were found in 3.1% (p = 0.01) and 6.1% (p = 0.01) of the pooled DNA in the presence of sense or AS ssODNs, respectively (Fig. 4C). In contrast, with Cas9D10A nickase, evidence for template-based gene editing was only observed when combined with an AS ssODN, although at a much lower efficiency (0.9%, p = 0.001) in comparison to DSB repair with a ssODN. Templated repair with the sense ssODN following Cas9 nickase activity could not be observed by RFLP.

To determine the functional consequences of gene editing, treated mESCs were subjected to 12.5 nM MMC in a clonal survival assay (Fig. 4D). In contrast to the mouse fibroblasts described above, MMC selection of mESCs was far more discriminatory, as mock treated cells almost completely failed to form colonies in the presence of MMC. After formation of Cas9-mediated DSBs, 27.8% (p = 0.004) or 35.6% (p = 0.0003) of the ESC colonies had gained resistance to MMC following error-prone end joining or templated repair with the sense ssODN or antisense ssODN, respectively. The nickase treated populations with the sense and AS ssODN showed respectively 0.9% (p = 0.07) and 2.0% (p = 0.025) clonal survival in the presence of MMC. These data indicate that gene editing was capable of successfully restoring *Fancf* gene function, with the antisense ssODN giving significantly higher MMC cell survival after Cas9 DSB formation in comparison to the sense ssODN (p = 0.01). Moreover, Cas9 DSB formation and error prone repair resulted in at least 17-fold higher clonal survival levels than Cas9 nick formation (p ≤ 0.0025) (Fig. 4D).

### Detection of templated gene editing in single cell derived mESC clones

To assess gene editing outcomes at the single cell level, mESC clones obtained without or after MMC selection (Fig. 4D) were analyzed by standard RFLP analysis and allele confirmation through Sanger sequencing. Positive *Kpn*I digests, indicative of template-based gene editing, were readily detectable in MMC-selected and non-selected clones (Table 2). Importantly, mock transfected cells with a non-targeting sgRNA and with the ssODNs never showed a positive *Kpn*I RFLP, indicating the ssODN by itself did not modify the *Fancf* locus at detectable frequencies (not shown). Without MMC exposure, 10.6% (sense ssODN) and 20% (AS ssODN) of the analyzed cell clones revealed template-based gene editing after Cas9 DSB formation (Table 2), which is almost twofold higher than expected from the frequency of *Kpn*I-digestible alleles in the cell pools (Fig. 4C). It seemed templated editing with the AS ssODN was more efficient, although this difference was not significant (one sided Fisher exact test (FET) p = 0.22). Remarkably, non-MMC selected cell clones obtained with the Cas9D10A nickase revealed much higher frequencies of templated-editing than expected from Fig. 4C: 10.4% with sense ssODN, 31.2% with AS ssODN (p = 0.03 FET) (Table 2).

**Table 2:** *Kpn*I RFLP analysis of mESC clones.

Cas9	ssODN	MMC	Analyzed number of clones	Positive *Kpn*I RFLP%	*Kpn*I homo/hemizygosity%
WT	Sense	−	47	10.6	4.3
	Sense	+	47	12.8	4.3
	AS	−	45	20.0	2.2
	AS	+	47	53.2	12.8
D10A	Sense	−	48	10.4	2.1
	Sense	+	5	80.0	0
	AS	−	48	31.2	0
	AS	+	22	81.8	0

*Kpn*I RFLP frequencies further increased in cell clones obtained in the presence of MMC, with the frequency of AS ssODN in combination with wtCas9 more than doubling (53.2%) and now significantly outcompeting the sense ssODN (12.8%; p = 0.002 FET). Even more strikingly, *Kpn*I RFLP frequencies in MMC-selected single cell-derived clones obtained following Cas9D10A activity reached 80.0% (with sense ssODN) and 81.8% (with AS ssODN). Thus, among single cell clones the percentages of template-corrected cells appeared much higher than observed in the treated cell pools (Fig. 4C). This observation indicates that the outgrowth of single cell ESC clones strongly benefits from an active FA pathway and hence provides a convenient selection for successful FA gene correction events.

In the majority of clones showing the *Kpn*I RFLP, only one allele had undergone templated repair of the double- or single-stranded break. After Cas9 DSB formation, 3 out of 92 analyzed clones (3.2%) appeared to be homozygous, showing only the templated allele (Table 2). This figure was only slightly higher after MMC selection: 8 out of 94 clones (8.5%). After introduction of a single-strand nick, only 1 out of 96 clones (1%) revealed templated-repair allele homozygosity, while none of 27 nickase exposed clones obtained after MMC selection were homozygous. Homozygosity for the templated-repair allele may indicate that both *Fancf* alleles in the mESCs were corrected. Alternatively, only one allele was corrected while the second allele sustained DNA changes that prevented PCR amplification in our assay.

Since the majority of clones showing the *Kpn*I RFLP appeared heterozygous, we sequenced *Fancf* genomic DNA fragments from individual clones in order to identify the status of the other allele (Suppl. Table 3). In 8 out of 9 clones with a positive *Kpn*I digest signature following Cas9 DSB formation, accurate template-mediated *Fancf* correction was identified (89%), while one also had an in-frame duplication in the template-repaired allele (Suppl. Table 3). The second *Fancf* allele in these clones was always affected by gene editing, with 8 clones harboring indels and one clone showing *Kpn*I homozygosity, possibly reflecting bi-allelic templated repair (Suppl. Table 3).

In contrast, mono-allelic gene modification dominated in clones obtained following Cas9D10A nickase activity. First, in all 20 clones with positive *Kpn*I RFLP, accurate templated repair of one allele was confirmed. In 19 of these 20 clones, the other *Fancf* allele remained unaffected while one clone had undergone bi-allelic templated repair or loss of heterozygosity (Suppl. Table 3).

In addition to *Kpn*I-positive clones, we also analyzed 19 Cas9D10A exposed clones that were *Kpn*I negative and not derived from MMC-exposed cultures. All had retained the homozygous mutant *Fancf* c.828insTAAA alleles except for one clone which revealed an open-reading-frame-restoring deletion of 7 nucleotides (Del 829–835 TAAAGTC). Finally, 5 RFLP *Kpn*I-negative/MMC-selected cell clones were sequenced. One of these clones appeared to contain a *Kpn*I site that was not detected by RFLP, while the others contained indels or partial HDR events and a nucleotide substitution that restored the *Fancf* ORF, with two clones showing wildtype *Fancf* alleles (Suppl. Table 3). The second *Fancf* allele in these 5 clones remained unchanged. Thus, in 25 out of 26 clones that were modified following Cas9D10A nickase activity by either accurate templated repair (21 clones) or error-prone repair, the genetic modification was confined to one allele, while the other allele remained unaffected. Only one clone had possibly undergone bi-allelic templated gene correction or loss of heterozygosity.

### Confirmation of FA pathway activity following gene editing

**Figure 5:**
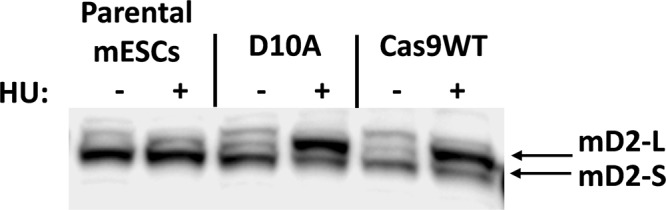
Activation of the FA pathway after CRISPR/Cas9 templated repair in mouse ES cells. Western blot to detect short and long forms of FANCD2 in parental and template corrected mESCs. Upon exposure to hydroxy urea (HU) the short (S) from of FANCD2 becomes the long (L) form due to the ligation of a mono-ubiquitin group mediated by a functional FA core complex. The ESC corrected by Cas9D10A revealed one template repaired allele and one c.828InsTAAA allele, while the Cas9 WT ESC appeared homozygous for templated repair.

While MMC resistance, *Kpn*I digestion, and sequence confirmation of gene correction in *Fancf* demonstrated that FA genes can be restored by gene editing, further proof of FA pathway reinstatement was obtained by studying FANCD2 mono-ubiquitination. Fig. 5 shows the FANCD2 status in FANCF-deficient parental mESCs and two independent mESC clones obtained after templated gene editing, with and without hydroxyurea (HU) exposure. The template-based gene edited cells showed robust induction of ubiquitinated FANCD2 after exposure to HU, confirming FA pathway functionality.

## Discussion

The correction of FA mutations by CRISPR/Cas9 gene editing in hematopoietic stem cells bears promise for a future treatment against bone marrow failure in patients. However, DNA repair deficiencies intrinsic to FA cells may hinder gene editing efficacy. In order to test the use of CRISPR/Cas9 in FA-deficient cells, we performed gene editing experiments in fibroblasts and embryonic stem cells derived from a *Fancf* mouse model with a pathogenic bi-allelic c.828insTAAA mutation (Fig. 1). Importantly, we found that targeting the *Fancf* c.828insTAAA mutation by CRISPR/Cas9 allowed functional gene correction via error-prone DNA end joining. This has not been previously assessed and may be a viable gene editing strategy to correct FA mutations involving small nucleotide alterations in cells with compromised HDR capacity. The efficiency of this strategy depended on the sgRNA that was used to direct Cas9 activity. As assessed by the clonal outgrowth of fibroblasts in the presence of MMC and sequence analysis of surviving clones, sgRNA + 1 performed better than sgRNA-14, likely because it allowed restoration of the ORF with only a few amino acid substitutions that were apparently tolerated. Functionality of divergent FA proteins has previously been documented in cross-species complementation assays^42,43^, mutagenesis experiments^37^, and by compensating gene alterations observed in patients with reverse mosaicism^15–17^.

We then assessed the feasibility of precise homology-directed gene editing by providing wildtype Cas9 with sgRNA + 1 and a 120 nt ssODN that deletes the TAAA insertion while simultaneously creating a *Kpn*I restriction site. By TIDER, *Kpn*I RFLP and MMC survival analysis, we compared the outcome of gene editing events in fibroblasts and mESCs (Suppl. Table 4 provides a summary of the results). While TIDER analysis indicated higher indel frequencies in fibroblasts than in mESCs (~72% versus ~46%), the frequencies of productive gene editing events (MMC surviving clones) and templated repair (sensitivity to *Kpn*I digestion) were similar in the two cell types (~30% and ~3–6%, respectively).

Considering the functions of FA proteins in replication fork stability^44,45^ and canonical homologous recombination^1,46^, we expected that mouse embryonic stem cells would be more suited to uncover potential gene editing deficiencies as rapidly proliferating mESC spend most of their time in S-phase and are highly proficient in HDR^41,47^. However, templated gene editing appeared equally effective in fibroblasts and mESCs. While these data provide proof of principle that ssODN templated repair is feasible in FA-deficient mouse ESCs, the modest efficiencies obtained (3–6%) suggest that the FA defect may compromise the efficacy of templated repair. Previously, we observed templated gene efficiencies of ≥40% in wildtype mESCs using a highly similar gene editing protocol^25^. Although this difference could be related to differential gene editing efficiencies at distinct loci, our observations are in line with the findings of Richardson *et al*. that FA pathway members promote efficient ssODN templated gene editing after DSB formation^29^.

Similar experiments were performed using Cas9D10A nickase. As expected, gene editing with Cas9D10A nickase resulted in lower indel frequencies in both cell types, while templated repair as judged from *Kpn*I RFLP levels were not significantly higher than mock control samples, except for antisense ssODN treated mESCs. Indeed, Cas9 nickase activity in combination with antisense ssODN resulted in a low 2% survival in mESCs, which was significantly above background levels. After single-strand break formation, templated repair with the sense ssODN has been proposed to follow single-strand DNA incorporation, while the antisense ssODN is a substrate for annealing driven strand synthesis (ADSS). Our results indicate the latter is more effective, in agreement with other reports^22^.

Besides looking at gene editing events in the pool of mESCs, we also analyzed individual mESC clones by *Kpn*I RFLP analyses and Sanger sequencing and made two striking observations.

First, correction of the FA defect conferred a strong proliferative advantage. Sense and antisense ssODN-templated repair events after DSB formation in unselected mESC clones amounted to 10.6% and 20.0%, respectively. These frequencies are 1.7 and 1.8-fold higher than the anticipated 6% and 12% based on *Kpn*I RFLP analyses in the mESC pool, and the assumption that mESCs are diploid and templated repair likely affects only one *Fancf* allele. This points towards a proliferation advantage for corrected cells in the clonal survival assay even without exposure to MMC. An even stronger proliferative advantage was observed in clones obtained in the presence of MMC: templated gene editing events had increased to 12.8% with sense and 53.2% with AS ssODNs. Sanger sequencing of mESC clones with positive *Kpn*I RFLP following wtCas9/ssODN exposure confirmed accurate templated repair events in 89% of the analyzed clones.

An even stronger effect was seen in mESC clones obtained after gene editing with Cas9D10A nickase. *Kpn*I RFLP analysis of mESC clones revealed a notable shift in the percentage of templated repair events, increasing from barely detectable in the pools up to 10.4% with the sense and 31.2% with the antisense ssODN. This implies a >30-fold enrichment for template-edited mESC clones during clonal expansion. In the presence of MMC, the percentage of template-repaired clones raised to even 80%. Together, these results indicate that even with a very low efficiency of templated repair of the FA defect, subsequent clonal outgrowth provides a strong selection for successfully corrected clones without the need of applying genotoxic stress to select for FA proficiency. This observation is consistent with the significant selection bias against *Fancf* c.828insTAAA homozygous mutant ESCs we previously observed when we attempted to establish mESC cultures from blastocysts and a severe proliferation disadvantage following the disruption of *FANCA* in human ESCs^48^.

Secondly, sequencing of *Kpn*I RFLP positive mESC clones, either obtained with wtCas9 or Cas9D10A, revealed that in most cases only one allele was modified by templated gene editing. With wtCas9, the second *Fancf* c.828insTAAA allele was almost always modified by small indels characteristic for error-prone repair. One clone may either have been subjected to bi-allelic templated repair, or the second *Fancf* allele sustained a large deletion preventing PCR amplification. In contrast, ESC clones obtained by templated gene editing following Cas9D10A nickase activity usually carried an undisrupted *Fancf* c.828insTAAA second allele, with only one clone carrying a bi-allelic templated repair signature. These results confirm previous observations that nick-mediated gene editing is far less mutagenic than DSB-mediated gene editing^10,21,23^. Besides templated repair, mutagenic repair after Cas9D10A activity was observed in four MMC-selected clones displaying deletions and/or partial templated repair events resulting in restoration of *Fancf* gene function. However, the other *Fancf* allele remained unaffected in these clones as well.

Since others have previously also documented successful HDR gene editing strategies to repair or compensate defects in *FANCA*, *FANCC*, *FANCD1*/*BRCA2* and *FANCI*, the combined data support that gene editing can be a promising therapeutic strategy to prevent onset of bone marrow failure in FA patients^27,30,32,33^. Moreover, Diez *et al*. were able to complement FANCA-deficient hematopoietic stem cells by ZFN-mediated safe harbor targeting despite their intensified stress response, and others have applied various techniques to perform gene editing in non-FA hematopoietic stem cells^31,49,50^. In the development of gene editing therapy against FA bone marrow failure, both *ex vivo* as well as *in vivo* strategies should be considered. While *ex vivo* gene editing offers the opportunity to extensively investigate on- and off-target effects, it also exposes FA-HSCs to stress-inducing *in vitro* culture conditions before gene correction can be accomplished. In contrast, *in vivo* gene editing requires the delivery of gene editing tools to HSCs within the bone marrow niche, where their quiescent nature may constrain templated repair. Nevertheless, both *ex vivo* and *in vivo* therapeutic strategies should be greatly supported by the proliferative advantage gained by FA corrected cells as observed in our experiments, in CD34^+^ cells, and in mosaic patients^17,31^. The development of the *Fancf*828InsTAAA mouse model and the documented efficacy of established gene editing tools in this study provide a solid basis to initiate pre-clinical experiments to assess the feasibility of *ex vivo* an *in vivo* gene editing in FA-deficient HSCs.

Naturally, therapeutic safety is a principal concern. In our assays we did detect low levels of off-target DNA alterations (~8%) in *Aatk* and *Apba1*, both loci being highly similar to the *Fancf* target site (Table 1). Moreover, sequence analysis revealed DNA integrations of the gene editing plasmid at the *Fancf* target site at low frequency. Hendel *et al*. have previously documented similar undesirable gene editing outcomes^51^. Notably, in our experiments Cas9 nuclease activity was boosted by transient puromycin selection, and in a therapeutic setting undesirable gene editing events can be avoided by careful titration of Cas9 ribonucleoprotein concentrations, implementing Cas9 variants with improved specificity, and applying synthetically modified sgRNAs^19,52–54^.

The pre-clinical development of therapeutic applications against FA is further supported by our findings that Cas9D10A nickase in mESCs provides high precision mono-allelic gene editing, with the initial low *Fancf* correction frequencies being negated by the competitive proliferative advantage of FA-corrected cells.

## Methods

### Establishing *Fancf* c.828insTAAA mutant cell lines

*Fancf* fibroblast derivation. Fibroblasts were derived from mouse ears. Ears were removed from euthanized mice with the desired genotype, rinsed in 70% EtOH, washed in PBS, and diced into small pieces. The individual ear pieces were placed on the bottom of a culture well (6 wells plate) and immersed in DMEM supplemented with 10% FCS under standard conditions. Fibroblasts were expanded over 10 passages to be considered immortalized.

Mouse embryonic stem cell derivation. Mouse embryonic stem cells (ESCs) with bi-allelic mutations were established after crossing heterozygous *Fancf* c.828insTAAA mice and harvesting blastocysts. Derivation and expansion of mESCs from blastocysts was performed as described^55^. Genotyping was performed as described in Suppl. Table 5. Statistical analysis on mESC frequencies was performed by Fisher Exact Test. Research with the *Fancf* c828.InsTAAA mouse model was approved by the laboratory animal ethics committee of The Netherlands Cancer Institute and all mouse handling and experiments were carried out following applicable guidelines and regulations.

### Fibroblast growth-inhibition test

A total of 3000 or 5000 cells were plated in 12 wells in duplicate. After 24 hours the cells were exposed to MMC at the concentrations of 0, 5, 10, 20, 40, and 80 nM. After growing for 6 days when untreated wells had reached confluency, the cells were fixed and stained with 2% Methylene Blue in 37% EtOH and subsequently rinsed in water and air-dried. The 12 well plates were photographed and converted to binary files. Positive surface area was quantified using ImageJ software to measure cell density. Non-treated wells were considered as 100% growth and relative growth of the cells in MMC treated was calculated. The growth inhibition test was performed twice. Statistical analysis was performed using the Student’s t-test.

### Construction of gene editing plasmids

Vectors expressing human codon optimized *Streptococcus pyogenes* Cas9 (px260, pX330) or Cas9D10A (pX335) were obtained from Addgene. The puromycin cassette of plasmid pX260 was amplified with Phusion polymerase (New England BioLabs) using primers described in Suppl. Table 5. The backbones of the pX330 and pX335 plasmids were linearized using two adjacent *Sma*I restriction sites (New England BioLabs) and the PCR product encoding the puromycin cassette was inserted by InFusion cloning (Clontech). Next, the *Fancf* target sequences were cloned into the single guide RNA (sgRNA) cassettes of pX330Puro and pX335Puro. The sgRNA expression cassettes were digested using *Bbs*I (New England BioLabs) and annealed oligonucleotides encoding sgRNA-14 and sgRNA + 1 were cloned in to the vectors by InFusion reactions (see Suppl. Table 5). All constructs were confirmed by Sanger sequencing.

### Fibroblast transfection and selection

*Fancf* mutant fibroblasts were seeded at 3 × 10^5^ per well in a 6-well plate and transfections were performed the next day with Lipofect LTX following manufacturer’s instructions (Invitrogen) using 5.9 μg pX330Puro_sgRNA-14, pX330Puro_sgRNA + 1, or pX330Puro_Mock DNA and 0.1 μg pEGFP-N1 plasmid (Clontech) DNA to monitor transfection efficiencies. The mock vectors actually encode an 18 nucleotide sgRNA without identical target sequence in the mouse reference genome (see Suppl. Table 5). In experiments with template DNA, 4.9 μg gene editing vector was applied with 1 μg single-strand oligonucleotide either in sense or AS orientation (see Suppl. Table 5). 24 hours after transfection the fibroblasts were exposed to puromycin at 1.8 μg/ml for 48 hours to select for transient vector uptake. Next, cell cultures were allowed to proliferate for 2 to 3 days and fibroblasts were harvested for gene editing analysis by Surveyor assay and clonal survival assays.

### Surveyor assay and detection of templated repair by restriction fragment length polymorphism

The gene edited region of the *Fancf* locus was amplified using Phusion polymerase (see Suppl. Table 5). PCR products were denatured and allowed to re-anneal to allow heteroduplex formation. The Surveyor assay was performed following manufacturer’s instructions (Transgenomic, UK) and gene editing frequencies were calculated as described^56^. In experiments with a template ssODN for homology-directed repair *Kpn*I-HF (New England Biolabs) digestion reactions were performed on PCR product generated with the same primers. In standard RFLP assays, digested PCR products were run on an agarose gel. For quantification of templated repair events in the cell pools, 5 or 10-fold diluted *Kpn*I digested PCR products were analyzed on a TapeStation 2200 (Agilent Technologies) using ScreenTapes D1000 or HSD1000. DNA concentrations were determined using manufacturer’s Analysis Software A.02.01. Templated repair events were calculated as percentage of digested DNA product against the total DNA concentration in the reaction, after background subtraction and normalization for *Kpn*I activity in a heterozygous control sample.

### Clonal Survival Assay

Fibroblasts subjected to gene editing were seeded at 3000 (Fig. 2D) or 2000 (Fig. 3D) cells per 10 cm culture dish. For each transfection condition 4 dishes were seeded with cells. MMC was added to two plates at a concentration of 15 nM (Figs. 2D) or 12.5 nM (Fig. 3D) MMC and cells were allowed to proliferate for approximately 10 days to support the formation of visible colonies. The colonies were fixed and stained with 2% Methylene Blue in 37% EtOH and plates were rinsed with water and air-dried. Colony counts were obtained manually or by using an automated colony counter (COL COUNT- Oxford Optronix), and the relative growth of MMC exposed cells was determined on the basis of colony counts observed in non-MMC-treated plates.

### *Fancf* gene editing analysis in MMC surviving fibroblast colonies

Fibroblast colonies that formed in the presence of MMC were collected and expanded in 96 well plates. Cells were harvested, centrifuged for 5 minutes at 3000 rpm to remove media and dissolved in 100 μl Direct PCR Tail Lysis solution (VIAGEN) with Proteinase K (Sigma-Aldrich). Cell lysis took place overnight at 55° C followed by a 15 minutes 82° C heat inactivation step. 1 μl cell lysate was added to 24 μl PCR mix. PCR assays were performed as for the Surveyor assay, products were cloned into the pJET vector (ThermoScientific) and transformed into *E*. *coli* DH5α (New England Biolabs). Five to ten random colonies were picked from each bacterial plate representing *Fancf* alleles from a single cell clone. PJet inserts were amplified and Sanger sequencing was performed to assess gene editing-mediated DNA alterations in *Fancf* (Suppl. Table 5).

### Gene editing in mouse embryonic stem cells

The gene editing plasmids and HDR template used to edit the mutant *Fancf* locus in fibroblasts were also applied in mouse ESCs. Transfections were performed 24 hours after seeding 3*10^5^ cells on laminin-coated (Sigma-Aldrich) 6 well plates. ESCs cells in 2i media were transfected using Trans-IT LT-1 reagent according to manufacturer’s instructions (Mirus) with 2.5 μg total DNA; 1.9 μg gene editing plasmid, 0.1 μg pEGFP-N1, and 0.5 μg ssODN. 24 hours after transfection cells were selected for transient vector uptake by exposure to puromycin at 0.9 μg/ml for 48 hours. Cells were allowed to recover from puromycin selection for approximately 3–5 days after which the cells were used for gene editing analysis or clonal survival.

### Gene editing frequencies determined by TIDER and TIDE

The *Fancf* locus was amplified and sequenced as described in Suppl. Table 5. TIDER quantification of small indels and occurrence of *Fancf* wildtype sequences were performed at https://tider-calculator.nki.nl, following standard settings^40^, using sgRNA + 1 reference, a mock control sequence chromatogram as control sample, and a wildtype *Fancf* sequence chromatogram as reference. Depicted values were derived from TIDER analyses with R^2^ values ≥ 0.9.

Putative off-target sites for sgRNA + 1 were identified using the http://crispr.mit.edu algorithm. Three top intragenic off-targets loci were analyzed (Table 1) after PCR amplification and Sanger sequencing on DNA from transfected cell pools and compared to matching sequence chromatograms obtained from parental ESC DNA and indel formation by TIDE using standard settings (https://tide.deskgen.com)^57^. Background indel levels assigned by TIDE were obtained by comparing *Fancf* on-target sequence chromatograms from mock transfected ESC pools with sequence chromatogram of the parental ESC cell line. Significant differences between indel levels were determined by Student’s T-Test.

### Mouse ES clonal survival assays

For each gene editing condition, 500 ES cells were seeded in quadruplicate in a 6-well format gelatinized culture plate. Two out of four wells were exposed to 12.5 nM MMC and incubated for approximately 10 days. The number of ES cell colonies were determined by placing the well plate on a transparent overhead sheet with a 0.5 cm^2^ grid and counting colonies using a light microscope (Zeiss Axio Vert.A1). Single cell-derived mouse ESC colonies were isolated from untreated and MMC treated wells and expanded for *Fancf* allele analysis. *Fancf* PCR and sequencing were performed as described above.

### FANCD2 protein detection

Mouse ESCs were harvested after exposure to 1 mM hydroxy urea (HU) for 24 hours. Western blotting was performed using 3–8% Tris-Acetate gels (ThermoFisherScientific). The antibody against mouse FANCD2 was kindly provided by K. J. Patel.

The datasets generated during the current study are available from the corresponding authors on reasonable request.

## Electronic supplementary material

Supplementary Info

## References

[1] Van de Vrugt, H. J. & Grompe, M. Fanconi anemia in *Epstein’s Inborn* Errors *of Development* (Eds Erickson, R. P. & Wynshaw-Boris A. J.) 1133–1139 (Oxford University Press, 2016).

[2] Park JY (2016). Complementation of hypersensitivity to DNA interstrand crosslinking agents demonstrates that XRCC2 is a Fanconi anaemia gene. J. Med. Genet..

[3] Bluteau D (2016). Biallelic inactivation of *REV7* is associated with Fanconi anemia. J. Clin. Invest..

[4] Knies K (2017). Biallelic mutations in the ubiquitin ligase *RFWD3* cause Fanconi anemia. J. Clin. Invest..

[5] Anur P (2016). Late effects in patients with Fanconi anemia following allogeneic hematopoietic stem cell transplantation from alternative donors. Bone Marrow Transplant..

[6] Mehta, P. A. *et al*. Radiation-free, alternative-donor HCT for Fanconi anemia patients: results from a prospective multi-institutional study. *Blood.* **129**, 2308–2316 (2017). 10.1182/blood-2016-09-743112 PMC5766838 28179273

[7] Ebens CL, DeFor TE, Tryon R, Wagner JE, MacMillan ML (2018). Comparable outcomes after HLA-matched sibling and alternative donor hematopoietic cell transplantation for children with Fanconi anemia and severe aplastic anemia. Biol. Blood Marrow Transplant..

[8] Ebens CL, MacMillan ML, Wagner JE (2017). Hematopoietic cell transplantation in Fanconi anemia: current evidence, challenges and recommendations. Expert Rev. Hematol..

[9] Río P (2017). Engraftment and *in vivo* proliferation advantage of gene-corrected mobilized CD34^+^cells from Fanconi anemia patients. Blood.

[10] Mali P (2013). RNA-guided human genome engineering via Cas9. Science.

[11] Jinek M (2012). A programmable dual-RNA-guided DNA endonuclease in adaptive bacterial immunity. Science.

[12] Cong L (2013). Multiplex genome engineering using CRISPR/Cas systems. Science.

[13] Doudna, J. A. & Charpentier, E. The new frontier of genome engineering with CRISPR-Cas9. *Science* **346** (2014). 10.1126/science.1258096 25430774

[14] Lo Ten Foe JR (1997). Somatic mosaicism in Fanconi anemia: Molecular basis and clinical significance. Eur. J. Hum. Genet..

[15] Waisfisz Q (1999). Spontaneous functional correction of homozygous Fanconi anaemia alleles reveals novel mechanistic basis for reverse mosaicism. Nat. Genet..

[16] Gross M (2002). Reverse mosaicism in Fanconi anemia: Natural gene therapy via molecular self-correction. Cytogenet. Genome Res..

[17] Mankad A (2006). Natural gene therapy in monozygotic twins with Fanconi anemia. Blood.

[18] Asur RS (2018). Somatic mosaicism of an intragenic *FANCB* duplication in both fibroblast and peripheral blood cells observed in a Fanconi anemia patient leads to milder phenotype. Mol. Genet. Genomic Med..

[19] Hu JH (2018). Evolved Cas9 variants with broad PAM compatibility and high DNA specificity. Nature.

[20] Richardson CD, Ray GJ, DeWitt MA, Curie GL, Corn JE (2016). Enhancing homology-directed genome editing by catalytically active and inactive CRISPR-Cas9 using asymmetric donor DNA. Nat. Biotechnol..

[21] Davis L, Maizels N (2014). Homology-directed repair of DNA nicks via pathways distinct from canonical double-strand break repair. Proc. Natl. Acad. Sci..

[22] Davis L, Maizels N (2016). Two distinct pathways support gene correction by single-stranded donors at DNA nicks. Cell Rep..

[23] Kan Y, Ruis B, Takasugi T, Hendrickson EA (2017). Mechanisms of precise genome editing using oligonucleotide donors. Genome Res..

[24] Fei S, Knut S (2017). Optimizing the DNA donor template for homology directed repair of double strand breaks. Mol. Ther. Nucleic Acid.

[25] Harmsen T, Klaasen S, van de Vrugt HJ, te Riele H (2018). DNA mismatch repair and oligonucleotide end-protection promote base-pair substitution distal from a CRISPR/Cas9-induced DNA break. Nucleic Acids Res..

[26] Fortini P, Ferretti C, Dogliotti E (2013). The response to DNA damage during differentiation: Pathways and consequences. Mutat. Res..

[27] Osborn MJ (2016). CRISPR/Cas9 Targeted Gene Editing and Cellular Engineering in Fanconi anemia. Stem Cells Dev..

[28] Jasin M, Haber JE (2016). The democratization of gene editing: Insights from site-specific cleavage and double-strand break repair. DNA Repair.

[29] Richardson CD (2018). CRISPR-Cas9 genome editing in human cells works via the Fanconi anemia pathway. Nat. Genet..

[30] Río P (2014). Targeted gene therapy and cell reprogramming in Fanconi anemia. EMBO Mol. Med..

[31] Diez, B. *et al*. Therapeutic gene editing in CD34^+^ hematopoietic progenitors from Fanconi anemia patients. *EMBO Mol*. *Med*. e201707540 (2017). 10.15252/emmm.201707540 PMC5666315 28899930

[32] Osborn MJ (2015). Fanconi Anemia Gene Editing by the CRISPR/Cas9 System. Hum. Gene Ther..

[33] Skvarova Kramarzova, K. *et al*. CRISPR/Cas9-mediated correction of the *FANCD1* gene in primary patient cells. *Int*. *J*. *Mol*. *Sci*. **18** (2017). 10.3390/ijms18061269 PMC5486091 28613254

[34] Dekker M (2006). Effective oligonucleotide-mediated gene disruption in ES cells lacking the mismatch repair protein MSH3. Gene Ther..

[35] Kowal P, Gurtan AM, Stuckert P, D’Andrea AD, Ellenberger T (2007). Structural determinants of human FANCF protein that function in the assembly of a DNA damage signaling complex. J. Biol. Chem..

[36] Deans AJ, West SC (2009). FANCM Connects the genome instability disorders Bloom’s syndrome and Fanconi anemia. Mol. Cell.

[37] Léveillé F (2004). The Fanconi anemia gene product FANCF is a flexible adaptor protein. J. Biol. Chem..

[38] Auerbach, A. D., Smogorzewska, A. & Francis L. Fanconi Anemia Mutation Database. http://www2.rockefeller.edu/fanconi/ (2018).

[39] Todardo and Green. Quantitative studies of the growth of mouse embryo cells in culture and their development into established lines. *J*. *Cell Biol*. **17**, 299–313 (1963). 10.1083/jcb.17.2.299 PMC2106200 13985244

[40] Brinkman, E. K. *et al*. Easy quantification of template-directed CRISPR / Cas9 editing. Preprint at: https://www.biorxiv.org/content/early/2017/11/13/218156 (2017).

[41] Te Riele H, Maandag ER, Berns A (1992). Highly efficient gene targeting in embryonic stem cells through homologous recombination with isogenic DNA constructs. Proc. Natl. Acad. Sci. USA.

[42] Van de Vrugt HJ (2000). Cloning and characterization of murine fanconi anemia group A gene: Fanca protein is expressed in lymphoid tissues, testis, and ovary. Mamm. Genome.

[43] Van de Vrugt HJ (2002). Characterization, expression and complex formation of the murine Fanconi anaemia gene product Fancg. Genes to Cells.

[44] Schlacher K, Wu H, Jasin M (2012). A Distinct replication frork protection pathway connects Fanconi anemia tumor suppressors to RAD51-BRCA1/2. Cancer Cell.

[45] Lachaud C (2016). Ubiquitinated Fancd2 recruits Fan1 to stalled replication forks to prevent genome instability. Science..

[46] Jasin M, Rothstein R (2013). Repair of strand breaks by homologous recombination. Cold Spring Harb. Perspect. Biol..

[47] Ahuja AK (2016). A short G1 phase imposes constitutive replication stress and fork remodelling in mouse embryonic stem cells. Nat. Commun..

[48] Vanuytsel K (2014). *FANCA* knockout in human embryonic stem cells causes a severe growth disadvantage. Stem Cell Res..

[49] Ceccaldi R (2012). Bone marrow failure in Fanconi anemia is triggered by an exacerbated p53/p21 DNA damage response that impairs hematopoietic stem and progenitor cells. Cell Stem Cell.

[50] Dever DP, Porteus MH (2017). The changing landscape of gene editing in hematopoietic stem cells: A step towards Cas9 clinical translation. Curr. Opin. Hematol..

[51] Hendel A (2014). Quantifying genome-editing outcomes at endogenous loci with SMRT sequencing. Cell Rep..

[52] Casini A (2018). A highly specific SpCas9 variant is identified by *in vivo* screening in yeast. Nat. Biotechnol..

[53] Cromwell CR (2018). Incorporation of bridged nucleic acids into CRISPR RNAs improves Cas9 endonuclease specificity. Nat. Commun..

[54] Dewitt M, Corn JE, Carroll D (2017). Genome editing via delivery of Cas9 ribonucleoprotein. Methods.

[55] Huijbers IJ (2015). Using the GEMM-ESC strategy to study gene function in mouse models. Nat. Protoc..

[56] Guschin, D. Y. *et al*. A rapid and general assay for monitoring endogenous gene modification engineered zinc finger proteins. In *Engineered Zinc Finger Proteins* (eds Mackay, J. P. & Segal, D. J.) 247–256 (Springer, 2010). 10.1007/978-1-60761-753-2_15 20680839

[57] Brinkman EK, Chen T, Amendola M, Van Steensel B (2014). Easy quantitative assessment of genome editing by sequence trace decomposition. Nucleic Acids Res..

