# Supplementary material for: Effective CRISPR/Cas9-mediated correction of a Fanconi anemia defect by error-prone end joining or templated repair
Source: Sci Rep. 2019 Jan 25;9:768. doi: 10.1038/s41598-018-36506-w (PMC6347620; doi:10.1038/s41598-018-36506-w)
Supplement: Supplementary file 1 — Supplementary Info [file 41598_2018_36506_MOESM1_ESM.pdf]

Effective CRISPR/Cas9-mediated correction of a Fanconi anemia defect by error-prone end joining or templated repair

Henri J. van de Vrugt[1,2*], Tim Harmsen[1], Joey Riepsaame[1, 4], Georgina Alexantya[1], Saskia E. van Mil[2], Yne de Vries[2], Rahmen Bin Ali[3], Ivo J. Huijbers[3], Josephine C. Dorsman[2], Rob M.F. Wolthuis[2], Hein te Riele[1,2*].

1. Division of Tumor Biology and Immunology, The Netherlands Cancer Institute, Plesmanlaan 121, 1066 CX Amsterdam, The Netherlands.

2. Oncogenetics, Department of Clinical Genetics, Cancer Center Amsterdam, Amsterdam University Medical Centers, De Boelelaan 1118, 1081 HV Amsterdam, The Netherlands.

3. Mouse Clinic for Cancer and Aging research (MCCA) Transgenic Facility, The Netherlands Cancer Institute, Plesmanlaan 121, 1066 CX Amsterdam, The Netherlands.

4. Genome Engineering Oxford, Sir William Dunn School of Pathology, University of Oxford South Parks Road, OX1 3RE, Oxford, UK.

* corresponding authors

Supplemental Material

Supplemental text

Additional gene information of predicted off target sites sgRNA+1

Apoptosis-associated tyrosine kinase (*Aatk*)[#]
SgRNA+1 targets *Aatk* on mouse chromosome 11 at position 120,008,916 in intron 13. *Aatk* encodes a tyrosine kinase that is expressed during apoptosis. *Aatk* expression was detected in several tissues and is observed in a subset of hematopoietic (progenitor) cells, although a gene function has been proposed in neuronal differentiation. Expression was also detected in mouse embryonic fibroblasts, but has not been reported in mouse embryonic stem cells.

Podocalyxin-like 2 (*Podxl2*) [#]
SgRNA+1 targets *Podxl2* on mouse chromosome 6 at position 88,845,039, just 19 nucleotides downstream of the splice donor of intron 6. *Podxl2* encodes a surface transmembrane protein and is a member of the CD34 family. *Podxl2* expression is observed in several tissues and cell types, including mouse embryonic stem cells and fibroblasts, but has not been reported in hematopoietic progenitors. The Podxl2 protein is functionally involved in the interaction between vascular surfaces and leukocytes.

Amyloid-beta A4 precursor protein-binding family A member 1 (*Apba1*) [#]
sgRNA+1 targets *Apba1* on mouse chromosome 19 at position 23,948,003 in exon 13. *Apba1* encodes a protein that is a member of the X11 family of adapter proteins implicated in the formation of multiprotein complexes and is potentially important for adhesion and vesicle exocytosis of neuronal cells. Expression of *Apba1* appears predominantly in neuro-endocrine tissues. However, other studies have reported *Abpa1* expression in mouse embryonic stem cells, embryonic fibroblasts, and hematopoietic cells.

[#] The presented information was obtained from www.ensembl.org using version GRCm38.p6 of the mouse reference genome. Data on gene function and expression data were obtained from www.uniprot.org and the gene expression atlas at www.ebi.ac.uk/gxa/home.

Supplemental Table 1. Birth frequencies of *Fancf* c.828InTAAA mice from heterozygous breeding pairs

Genotype	%
WT	34
HET	41
MUT	25

Frequencies are based on 5 litters with a total of 44 pups

Supplemental Table 2A. Distinct *Fancf* alleles identified in mouse fibroblasts transfected with px330Puro sgRNA-14

Allele	DNA Sequence	Protein Sequence	Clones [#1]	Allelic Frequencies [#2]
1	828 InsTAAA (parental)	278 stop	3	8.8
2	814 InsC	270+22 stop	2	5.9
3	Del 805-811 TATGATC + 805 InsCA	268+22 stop	1	2.9
4	Del 805-814 TATGATCTGC	269-270 delYD + 271-276 LQKGAW>KRVPGK	1	2.9
5	Del 807-813 TGATCTG	270 delD + 271-276 LQKGAW>KRVPGK	1	2.9
6	Del 808-812 GATCT	269+21 stop	3	8.8
7	Del 808-814 GATCTGC	270 delD + 271-276 LQKGAW>KRVPGK	2	5.9
8	Del 808-816 GATCTGCAA	269 + 4 stop	1	2.9
9	Del 808-817 GATCTGCAAA	270-271 del DL + 272-276 QKGAW>RVPGK	2	5.9
10	Del 809-813 ATCTG	269+21 stop	2	5.9
11	Del 809-814 ATCTGC	269+5 stop	2	5.9
12	Del 810-813 TCTG	271-276 LQKGAW>KRVPGK	2	5.9
13	Del 810-814 TCTGC	269+21 stop	3	8.8
14	Del 810-818 TCTGCAAAA	269 + 4 stop	1	2.9
15	Del 811-813 CTG	270+5 stop	1	2.9
16	Del 811-814 CTGC	271-276 LQKGAW>KRVPGK	1	2.9
17	Del 812-815 TGCA	271-276 LQKGAW>QRVPGK	1	2.9
18	Del 812-817 TGCAAA & 811 Ins 70 nt [#3]	270+3 stop	1	2.9
19	Del 813-814 GC	271+20 stop	1	2.9
20	Del 813-815 GCA	271+4 stop	1	2.9
21	Del 817-818 AA	271+20 stop	2	5.9

#1: number of clones that contained indicated allelic variant of *Fancf.* #2: In total 34 *Fancf* alleles were identified in 12 fibroblast clones. #3: insertion identified as nucleotides 520 -589 of the *Beta-lactamase* coding sequence in the pX330Puro plasmid. Restored ORFs are indicated by grey background

Supplemental Table 2B. Distinct *Fancf* alleles identified in mouse fibroblasts transfected with px330Puro sgRNA+1

Allele	DNA Sequence	Protein Sequence	Clones #1	Allelic Frequencies #2
1	828 InsTAAA (parental)	278 stop	3	4.2%
2	830 Ins 113nt #3	278+17 stop	1	1.4%
3	830 Ins 29nt #4	279 Ins SWKRWTAPPRNK	1	1.4%
4	830 Ins A	278 stop	1	1.4%
5	830 Ins AA	278 stop	1	1.4%
6	830 Ins C	278+16 stop	1	1.4%
7	830 Ins CA	279 Ins SK	2	2.8%
8	830 Ins T	278+16 stop	1	1.4%
9	830 Ins TA	279 Ins YK	1	1.4%
10	832 Ins 37nt #5	278 stop	1	1.4%
11	Del 814-828 CAAAAGGGTGCCTGGT 830 Ins TA	271 stop	1	1.4%
12	Del 818-832 GGGTGCCTGGTAAA	275+14 stop	1	1.4%
13	Del 826-830 TGGTA	277+15 stop	2	2.8%
14	Del 826-834 TGGTAAAGT	277+59 stop	1	1.4%
15	Del 826-836 TGGTAAAGTCC	277+13 stop	1	1.4%
16	Del 828-829 GT 830 Ins A	277 stop	2	2.8%
17	Del 829 T	279 Ins K	9	12.5%
18	Del 829-830 TA	278+15 stop	1	1.4%
19	Del 829-832 TAAA	WT *Fancf*	2	2.8%
20	Del 829-833 TAAAG	278+14 stop	1	1.4%
21	Del 829-845 TAAAGTCCCTACACAGA	278+10 stop	1	1.4%
22	Del 830 A	278 stop	18	25.0%
23	Del 830-831 AA	278 stop	3	4.2%
24	Del 830-831 AA Ins830 T	279 Ins L	3	4.2%
25	Del 830-832 AAA	278+60 stop	1	1.4%
26	Del 830-833 AAAG	279 V>F	3	4.2%
27	Del 830-834 AAAGT	278+14 stop	2	2.8%
28	Del 830-836 AAAGTCC	279 V>S del280 P	1	1.4%
29	Del 831-834 AAGT	279 V>Y	4	5.6%
30	Del 831-835 AAGTC	278+14 stop	1	1.4%
31	Del 834-837 GTCC	278 stop	1	1.4%

#1: number of clones that contained indicated allelic variant of *Fancf*, #2: In total 72 *Fancf* alleles were identified in 27 fibroblast clones. #3: insertion identified as nucleotides 36 -148 of the *Beta-lactamase* coding sequence in the pX330Puro plasmid. #4: insertion identified as nucleotides 1254 - 1282 of the Cas9 coding sequence in the px330Puro plasmid. #5: Nucleotides 1-34 of the insertion are identical to mouse chromosome 7: nucleotides 27,623,159-27,623,195 encoding for *Akt2*. Restored ORFs are indicated by grey background

Supplemental Table 3. Identified *Fancf* alleles in template gene edited mouse ES clones applying sgRNA+1

Fancf alleles identified in mouse ES cell clones after gene editing with sgRNA+1 and repair.

Cas9 wild-type

Clone counts	RFLP	ssODN	MMC	DNA Sequence: Allele 1	DNA Sequence: Allele 2	Predicted Protein Sequence: Allele 1	Predicted Protein Sequence: Allele 2
2	*Kpn* I	Sense	+/-	HDR	Del 830-831 AA	..KGAWVPTQHED..	..KGAW*
1	*Kpn* I	Sense	-	HDR	Del 829-833 TAAA / 836InsA / 840InsC	..KGAWVPTQHED..	..KGAWSPTRWRTCPGKSCA*
1	*Kpn* I	Sense	+	HDR	HDR / Del 837-840 CTAC	..KGAWVPTQHED..	..KGAWVHRWRTCPGKSCA*
1	*Kpn* I	Sense	+	HDR	Del 830 A	..KGAWVPTQHED..	..KGAW*
1	*Kpn* I	Sense	+	HDR	Ins 830 C	..KGAWVPTQHED..	..KGAWSKSLHRWRTCPGKSCA*
1	*Kpn* I	Sense	+	HDR / Dup 807-824 TGATCTGCAAAAGGGTG	Del 823-834 GCCTGGTAAAGT	..KGADLCKGAWVPTQHED..	..KGPYRWRTCPGKSCA*
1	*Kpn* I	A.S.	+	HDR	Del 828-829 GT	..KGAWVPTQHED..	..KGAWKYLHRWRTCPGKSCA*
1	*Kpn* I *	A.S.	+	HDR	n.d.	..KGAWVPTQHED..	n.d.

Cas9D10A

Clone counts	RFLP	ssODN	MMC	DNA Sequence: Allele 1	DNA Sequence: Allele 2	Predicted Protein Sequence: Allele 1	Predicted Protein Sequence: Allele 2
19	*Kpn* I	Sense / A.S.	+/-	HDR	Parental	..KGAWVPTQHED..	..KGAW*
1	*Kpn* I *	Sense	-	HDR	n.d.	..KGAWVPTQHED..	n.d.
18	-	Sense / A.S.	-	Parental	Parental	..KGAW*	..KGAW*
1	-	Sense	-	Del 829-835 TAAAGTC	Parental	..KGAW-PTQHED..	..KGAW*
1	-	A.S.	+	HDR	Parental	..KGAWVPTQHED..	..KGAW*
1	-	Sense	+	HDR / Del 836-842 CCTACAC	Parental	..KGAWV--QHED..	..KGAW*
1	-	A.S.	+	829T>G / del832-835 AGTC	Parental	..KGAWDPTQHED..	..KGAW*
2	-	A.S.	+	W.T. (Del 829-832 TAAA)	Parental	..KGAWVPTQHED..	..KGAW*

A total of 9 clones edited with wildtype Cas9 were analyzed by sequencing to confirm the presence of the *Kpn*I site. A total of 44 clones exposed to Cas9D10A were analyzed to document allelic frequencies. Note that clones selected in the presence of MMC always carried at least one functionally corrected *Fancf* allele. Gene editing with either wildtype Cas9 or Cas9D10A differentially affects the second *Fancf* allele, with 100% of the wtCas9 treated clones showing InDels, while among 21 templated repaired Cas9D10A treated clones only 1 clone presented evidence that the second allele was affected, while all other clones (95%) maintained an unaffected parental allele (p=0.001 Fisher exact test). HDR: homology-directed repair using the ssODN as template, resulting into elimination of the c.828InsTAAA mutation (del 829-832) and introduction of the *Kpn*I recognition site 835C>A. A.S.: anti-sense. *Kpn*I * : RFLP detected only one allele, in which the *Kpn*I site was present. +/-: represents data from clones that were either selected by MMC, or were taken from non-MMC treated control wells. Parental: refers to the original *Fancf* c.828InsTAAA allele present in the cells before gene editing. W.T. = wildtype *Fancf* sequence. n.d. = not detected, implying homozygous HDR or second allele carries a big deletion. [#] One discrepancy between RFLP analysis and sequence analysis was observed among 53 samples: *Kpn*I was not detected by RFLP but was present during sequence analysis. Aberrant protein sequences indicated in red.

Supplemental Table 4. Summary of gene editing, RFLP, and clonal survival frequencies*

Strategy		Fibroblast pool			mESC pool			mESC clones		
								-MMC	+MMC	
		TIDER	*Kpn*I	MMC	TIDER	*Kpn*I	MMC	*Kpn*I	*Kpn*I	Sequencing
Wt Cas9	sense	76.1	2.9	30	44.1	3.1	27.8	10.6	12.8	HDR + indels other allele
	AS	67.7	2.9	30	47.8	6.1	35.6	20.0	53.2	
Cas9 D10A	sense	5.0	0.9	12	0.7	0	0.9	10.4	80.0	HDR + non-modified allele
	AS	15.0	0.7	0	3.5	0.9	2.0	31.2	81.8	

*Numbers indicate percentages

Supplemental Table 5. Applied oligonucleotides

Experiment	Orientation and purpose	Oligonucleotide sequence 5' - 3'
Genotyping	Wildtype *Fancf* forward	CTGCAAAAGGGTGCCTGGGT
	Mutant *Fancf* forward	GCAAAAGGGTGCCTGGTAAA
	Reverse primer	GAACCTCGAAATCTCCATCCAAGGCTTTGC
Puromycin cassette	Forward	GGTCGCCCGACGCCCTTACGTCCAGCCAAGCTTAG
	Reverse	TCACTGAGGCCGCCCCGTACTATGGTTGCTTTGAC
gRNA oligo's	sgRNA-14 sense	AAAGGACGAAACACCGCTGCACTATGATCTGCAAA
	sgRNA-14 antisense	TTCTAGCTCTAAAACTTTGCAGATCATAGTGCAGC
	sgRNA+1 sense	AAAGGACGAAACACCGTCTGTGTAGGGACTTTACC
	sgRNA+1 antisense	TTCTAGCTCTAAAACGGTAAAGTCCCTACACAGAC
	Mock target sequence	GGGTCTTCGAGAAGACCT
Template ssODNs	Sense	CCTGGACCTGCTCACAACGTGGGCCACGCGGCTGCACTATGATCTGCAAAAGGGTGCCTGGGTACCTACACAGATGGAGGACATGCCCTGGGAAGAGCTGTGCCTAAGGCTGCAGAGCTT
	Antisense	AAGCTCTGCAGCCTTAGGCACAGCTCTTCCCAGGGCATGTCCTCCATCTGTGTAGGTACCCAGGCACCCTTTTGCAGATCATAGTGCAGCCGCGTGGCCCACGTTGTGAGCAGGTCCAGG
RFLP and clone sequencing	*Fancf* forward and sequence primer	GTGCGGATGAGACACAGAAACTACTGCG
	Fancf reverse	AGCCTGGGAACTGAGAATCTACTCTAGCAC
	PJet forward	CGACTCACTATAGGGAGAGCGGC
	PJet reverse	AAGAACATCGATTTTCCATGGCAG
TIDE / TIDER	*Fancf* forward	CTGCTACGCGAGGAAGACC
	Fancf reverse	TCAGGGGCCAATTTTAAATGGT
	Fancf sequence primer	TGAGACACAGAAACTACTGC
	Aatk forward	CCAGTCCGTGGGAGGTATGA
	Aatk reverse	GCTTTGTGAGCATGTGGGGA
	Aatk sequence primer	GCAGAGGGACACCACGAATG
	Podxl2 forward	GGAGACCCAACAGGCATTCT
	Podxl2 reverse	TTGTGTCCCATAGCCCAGC
	Podxl2 sequence primer	GGATGAGGAGACAGGTATG
	Apba1 forward	CGTGAGCCCTCAGTTTGACT
	Apba1 reverse	GTCGGCCCCATTAGTGAGAG
	Apba1 sequence primer	ATAGCTGCCTGAGAAGGAC

Oligonucleotides were obtained from Sigma-Aldrich.

Supplemental Figure 1. Representative sequence chromatograms for TIDE on and off-target analysis

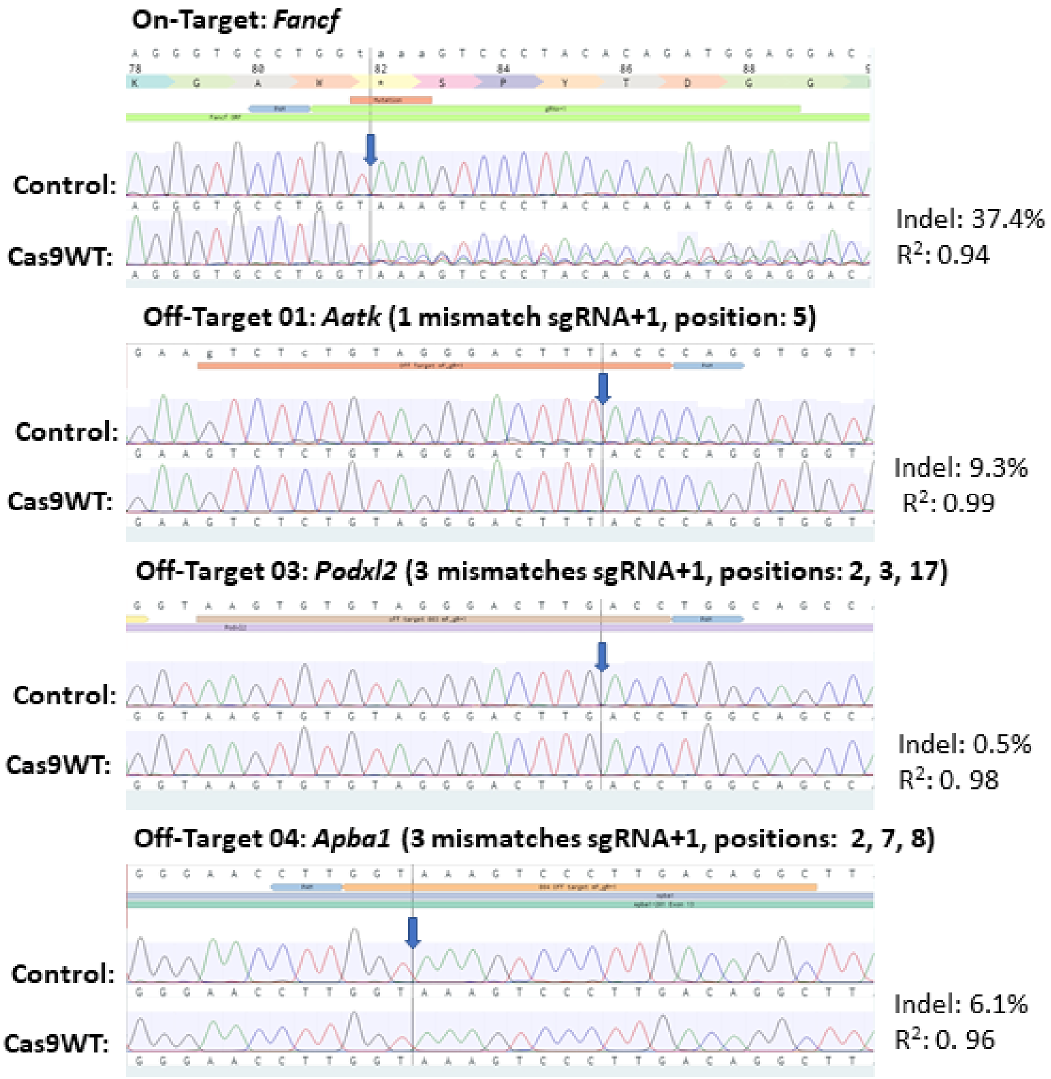

TIDE scores are presented on the right side of the figure. SgRNA recognition sequences and PAM motifs are shown. Blue arrows indicate predicted Cas9 DSB sites.

Supplemental Figure 2. FANCD2 western blot

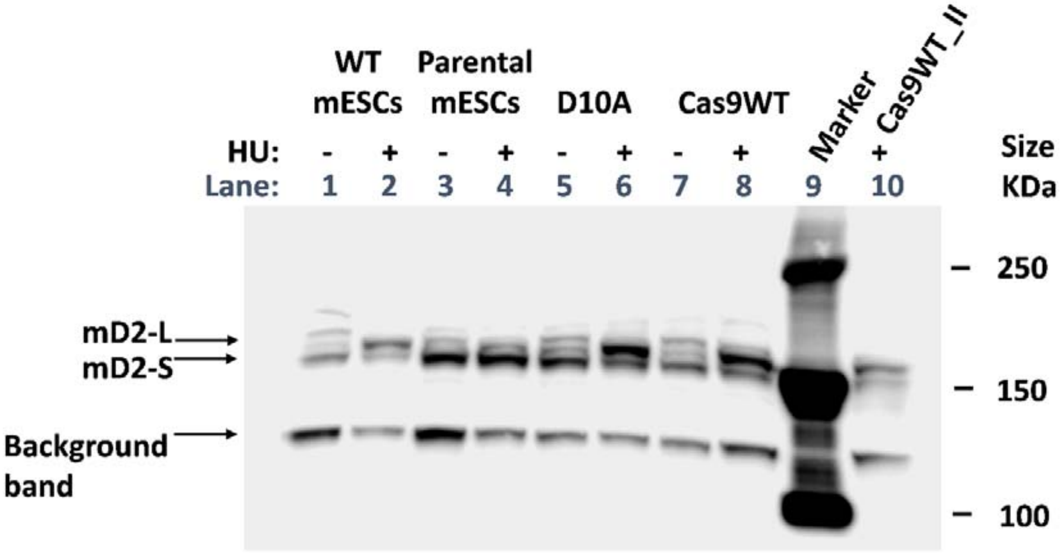

Full image of the cropped FANCD2 western blot image in manuscript figure 5. CRISPR/Cas9 gene editing of mutant *Fancf* restores FA pathway activity as observed by the activation of FANCD2 in the presence of hydroxy urea (HU). Lanes 1, 2: wildtype (WT) mouse embryonic stem cells (mESCs). Lanes 3, 4: parental mESCs mutant for *Fancf*. Lanes 5, 6: template edited mESC clone after exposure to Cas9 nickase (D10A). Lanes 7, 8: template edited mESC clone after exposure to wildtype Cas9. Lane 9: protein size marker. Lane 10: independent template edited mESC clone after exposure to wildtype Cas9. mD2-S: FANCD2-Short represents the non-ubiquitinated protein. mD2-L: FANCD2-Long represents the ubiquitinated protein.
